# Book Reviews

**Published:** 1897-10

**Authors:** 


### Book Reviews.

---

APPENDICITIS AND ITS SURGICAL TREATMENT, with a Report of Seventy-five Operated Cases. By HERMAN MYNTER, M. D. (Copenhagen), Professor of Operative and Clinical Surgery in Niagara University and Surgeon to the Sisters of Charity Hospital in Buffalo, N. Y. Octavo, pp. 303. Philadelphia: J. B. Lippincott Co. 1897.

So much has been written on the subject of appendicitis and it has been discussed so frequently in medical societies that it would seem as though all technical questions relating to its pathology, diagnosis and treatment must have been definitely settled by this time. Not so, however, nor is it likely that they will be determined during this day and generation. The conditions under which the disease manifests itself are so various, oftentimes so subtle and always so dangerous, that it is doubtful if definite lines of procedure can be agreed upon to meet all cases.

In the work before us we recognise the first attempt to cover, by a single treatise, the entire field occupied by appendicitis in its different phases. Professor Mynter leads off with a historical introduction that exhibits research and presents interesting data. While cases have been reported since 1759 (Mestivier), in which the appendix has been the offending cause of pus formations, it was not until 1888 (Sands) that a successful case of abdominal section, made during the first forty-eight hours of the malady, was reported. Most of the work between these two dates is merely a record of accident, conjecture, doubt and generally failure. During all this period it had generally been regarded as a medical disease and surgery was only resorted to when, late in the malady, pus formations were diagnosticated.

The first definite surgical discussion on appendicitis was held at the annual meeting of the medical society of the state of New York in February, 1891, at which time, as Mynter justly remarks, the surgical treatment suddenly came to the front. From this time forward there has been an increasing disposition on the part of those best informed to classify it as a surgical disease.

Mynter devotes a brief description to the anatomy of the appendix and its relations to the surrounding organs. He shows that it is an organ of unknown function, variable in length, breadth and thickness ; of inconstant position and of changeable relationship. Histologically its structure is much like that of the cecum, though rich in lymphoid cells, with two muscular coats and an ample vascular supply.

Under the head of etiology, Mynter proceeds to consider in consecutive order and with much attention to detail the various causes that may lead up to appendicitis. He makes two general heads, predisposing and exciting, under each of which he makes six subheads and discusses each with intelligence and precision. Contrary to the now generally received opinion he finds foreign bodies, such as cherry-stones, grape-seeds and the like, frequent exciting causes of appendicitis. Coprolites, a comprehensive and classic term for fecal concretions, are found, says Mynter, much more frequently than foreign bodies. They do not enter from the cecum, but are formed in the appendix itself and do not consist of feces except perhaps in the center, but of inspissated mucus and different salts colored with fecal matter. These concretions are recognised by surgeons as the most frequent causes of appendicitis and have in earlier times often been mistaken we believe for fruit seeds and stones, the heretofore traditional cause of appendicitis.

In the section devoted to pathology, Mynter groups some interesting facts. He shows how it was formerly the fashion to attribute deaths to inflammation of the bowels and peritonitis that were really caused by appendicitis. Every physician in medical practice in the anteoperative period is familiar with this fact. Even in Boston, according to Mynter, quoting from Richardson, between 1880 and 1884 inclusive, *i. e.*, before the operative period for appendicitis, 218 young men died from inflammation of the bowels, of which probably 194 were due to perforating appendicitis. During the same period 168 men and 155 women died of peritonitis at Copenhagen, which indicates—presuming that these were chiefly cases of appendicitis—that the disease is quite as prevalent abroad as in America, a view contrary to the generally accepted belief. Mynter continues the pathological discussion of the subject under the usual heads of ulcerative, perforative, infectious and gangrenous appendicitis, giving the views of some of the ablest pathologists on the various phases of the subject.

Under the head of symptomatology, our author gives a consecutive clinical picture of almost every manifestation of the malady. One of the most subtle and dangerous forms of appendicitis is that which develops without initial acute symptoms, and which steadily leads up to the point of danger while the victim is still walking about and even performing his daily duties. To persuade such a person to calmly lie down and have his abdomen opened on the supposition that he has an appendicitis, that only such an operation will cure, is not an easy thing to accomplish. Nevertheless, this has occasionally succeeded in saving a human life in patients intelligent enough to listen to scientific advice.

The complications and sequelæ of appendicitis furnish opportunity for skilful surgical knowledge and resourceful therapeusis. These questions are briefly but intelligently considered in this treatise. Under the head of diagnosis Mynter shows a completeness that is helpful. Nevertheless, there is always something in this subdivision that is wanting and doubtless always will remain so. Experienced surgeons are now generally agreed upon the propriety of operation as soon as diagnosis can be made ; they also agree that there are some cases in which diagnosis is shrouded in mystery and at best must be doubtful. We believe in such cases it is usually better to operate than to delay, for an aseptic incision made by a skilful surgeon is less dangerous than an unknown quantity lurking within the abdomen masked by a group of ill-defined symptoms.

Under prognosis Mynter reaffirms a surgical aphorism that it is doubtful in acute cases, as well as more unfavorable, the longer the operation is postponed. Under this head our author contrasts the difference between medical and surgical treatment and draws a picture less favorable to the former and increasingly favorable to the latter. As a matter of fact among the best informed medical men appendicitis has ceased to be regarded as a medical disease. The attending physician should, therefore, be prepared to operate at once, or summon a competent surgeon to do the work for him. Since death results in the main from gangrene, perforation or diffuse peritonitis, it follows that if operation can anticipate these grave consecutive conditions it will prove successful in the large majority of instances. There is no disease whose course is run more rapidly than appendicitis and the watchword should ever be—Time !

Under the head of treatment Mynter considers that in vogue in the several countries of Europe and America, quoting many of the leading operators on both sides of the ocean. He contrasts medical and surgical treatment in interesting juxtaposition and in his conclusions gives an intelligent consensus of the best surgical judgment of the day. He gives a résumé of his own statistics based on seventy-five operations personally conducted at the Sisters of Charity Hospital, and publishes the detailed history of these cases in part II. of the volume. These statistics end January 1, 1897, since which time, he informs the writer, he has operated on forty-seven cases in the same hospital, which affords an ample experience and entitles him to speak with authority.

This monograph was prepared to be submitted to Professor Mynter's Alma Mater, the University of Copenhagen, on which the degree of Doctor of Medicine was granted last July, twenty-six years after his graduation, an honor that carries dignity and has a meaning.

In conclusion, we feel justified in congratulating Professor Mynter on the preparation of such an intelligent and systematic treatise upon this important malady, one perhaps that interests the community more than any other at the present time. In this book will be found the opinions of many of the ablest surgeons in condensed form, with full references to the original text quoted. We miss, however, reference to a comprehensive discussion on the subject by the American Association of Obstetricians and Gynecologists, held at Toronto, September 19, 1894, which has been quoted on both sides of the Atlantic. As a final thought we may remark that this work of Mynter's is the most comprehensive treatise upon appendicitis yet published and it at once becomes a necessity for the student of the subject, whether he be physician, surgeon or teacher. The publishers have presented the book in attractive form.

TRANSACTIONS OF THE MEDICAL SOCIETY OF THE STATE OF NEW YORK for the year 1897. FREDERIC C. CURTIS, M. D., Secretary. Philadelphia: William J. Dornan, Printer. 1897.

This handsome volume of 584 pages is a record of the proceedings of one of the strongest and best organised state medical societies in the Union. We do not say this because we are of New York, but we are familiar with most of the societies, have access to their Transactions and believe we have stated a fact that will endure the test of comparison. This society is a delegate body, and while on its face its membership seems comparatively small it represents about 6,000 doctors. Its annual volumes contain the best thought of some of the most prominent members of the medical profession in the state and are eagerly sought as authoritative expositions of many important medical and surgical subjects. The book before us contains forty-six titles, besides the record of the proceedings, obituaries and lists of members in the various county medical societies. One of the most interesting as well as important, subjects discussed was on the Relation of impure water to disease and the cure and prevention of the latter. This embraces some twelve titles and includes almost every phase of the question at issue. Sanitarians and public health officers ought to obtain this volume, as it contains the latest and best literature relating to water contamination, the diseases resultant therefrom and the best methods of prevention.

The secretary has displayed great skill in the preparation of the volume, which involves an enormous amount of careful work.

---

TRANSACTIONS OF THE MEDICAL SOCIETY OF THE STATE OF NORTH CAROLINA. Forty-third Annual Meeting, held at Winston-Salem, N. C., May 12, 13 and 14, 1897. Wilmington: LeGwin Bros., Printers and Binders. 1896.

This society always has interesting meetings and its scientific work is of excellent quality, but it hardly does itself justice in publishing its transactions in the present form. The paper is poor and the letter-press inferior. It is, however, better to print in this way than not at all, for medical society work unpublished is lost. We look forward to the time when this sterling society will issue a volume second to none, for the *esprit de corps* of the profession in the old north state is well maintained and its work deserves preservation in form equal to the best. We cannot forget the stand taken by this society in regard to state examination for license and the high order of discipline its examining board maintains. The state board of health of North Carolina, too, is an efficient body that was created at the instance of this society. Both of these boards report to the state society, and their proceedings will be found printed in this volume.

---

A SYSTEM OF PRACTICAL THERAPEUTICS. By Eminent Authors. Edited by HOBART AMORY HARE, M. D., Professor of Therapeutics and Materia Medica in the Jefferson Medical College of Philadelphia. Volume IV. Octavo, 1,065 pages, with illustrations. Philadelphia and New York: Lea Brothers & Co., Publishers. 1897.

The popularity of the system of therapeutics edited by Professor Hare, that appeared about five years ago, has been widely attested. It is doubtful if any more valuable work on allied subjects has ever been printed in the English language.

The object of this volume is to bring the system forward to the present day. That it has covered the advances made in the interval is apparent upon careful examination. The contributors have been selected with judgment and the editor has performed his part judiciously. Professor Hare, himself, contributes articles on influenza, scarlet fever and measles, diabetes mellitus, and the intestinal parasites. His skill in the treatment of diseases pertaining to the department of internal medicine is well known, as a teacher he has but few equals, and as an author he is unsurpassed in the field of therapeutics.

Physicians who obtained the first volume of the series will be certain to possess this one, as it is a complement to the others and a necessary counterpart where completeness and advanced thought are desired.

---

A Handbook of Medical Climatology, Embodying Its Principles and Therapeutic Application, with Scientific Data of the Chief Health Resorts of the World. By S. Edwin Solly, M. D., M. R. C. S., late President of the American Climatological Association. In one 8vo volume of 470 pages, with engravings and colored plates. Philadelphia and New York: Lea Brothers & Co., Publishers. 1897.

It is becoming a recognised fact among scientific physicians that climate, quite as much as drugs, plays an important part in the treatment of many diseases. The aim of this work is to reduce climatology to a scientific basis as a therapeutic agent. Dr. Solly has done his work in a painstaking manner and has availed himself of government observations in reducing it to a scientific basis. The relief maps are an artistic study and the whole execution of the work is admirable. It gives a description of the principal health resorts, furnishes an analysis of their waters in many instances and supplies meteorological tables wherever essential. As a first attempt to treat the subject scientifically it is to be commended and is a volume that will easily find its way into the hands of every physician whose province is to prescribe a change of climate on an intelligent basis.

